# Painted Goby Larvae under High-CO_2_ Fail to Recognize Reef Sounds

**DOI:** 10.1371/journal.pone.0170838

**Published:** 2017-01-26

**Authors:** Joana M. Castro, M. Clara P. Amorim, Ana P. Oliveira, Emanuel J. Gonçalves, Philip L. Munday, Stephen D. Simpson, Ana M. Faria

**Affiliations:** 1 MARE–Marine and Environmental Sciences Centre, ISPA-Instituto Universitário, Lisbon, Portugal; 2 IPMA-Instituto Português do Mar e da Atmosfera, Algés, Portugal; 3 ARC Centre of Excellence for Coral Reef Studies, James Cook University, Townsville, Queensland, Australia; 4 Biosciences, College of Life and Environmental Sciences, University of Exeter, Exeter, United Kingdom; University of Auckland, NEW ZEALAND

## Abstract

Atmospheric CO_2_ levels have been increasing at an unprecedented rate due to anthropogenic activity. Consequently, ocean *p*CO_2_ is increasing and pH decreasing, affecting marine life, including fish. For many coastal marine fishes, selection of the adult habitat occurs at the end of the pelagic larval phase. Fish larvae use a range of sensory cues, including sound, for locating settlement habitat. This study tested the effect of elevated CO_2_ on the ability of settlement-stage temperate fish to use auditory cues from adult coastal reef habitats. Wild late larval stages of painted goby (*Pomatoschistus pictus*) were exposed to control *p*CO_2_ (532 μatm, pH 8.06) and high *p*CO_2_ (1503 μatm, pH 7.66) conditions, likely to occur in nearshore regions subjected to upwelling events by the end of the century, and tested in an auditory choice chamber for their preference or avoidance to nighttime reef recordings. Fish reared in control *p*CO_2_ conditions discriminated reef soundscapes and were attracted by reef recordings. This behaviour changed in fish reared in the high CO_2_ conditions, with settlement-stage larvae strongly avoiding reef recordings. This study provides evidence that ocean acidification might affect the auditory responses of larval stages of temperate reef fish species, with potentially significant impacts on their survival.

## Introduction

Ocean acidification, caused by the uptake of anthropogenic CO_2_ from the atmosphere, is increasingly recognized as a serious threat to marine ecosystems [1,2]. Exposure to high CO_2_ levels can affect physiological processes (e.g. [3,4]), calcification (e.g. [5,6]), development (e.g. [7,8]), and survival (e.g. [9,10]) of marine organisms, especially during their larval and juvenile stages. Furthermore, there is increasing evidence that larval behaviour can be disrupted by elevated CO_2_ levels (e.g. [11,12,13]), which may ultimately affect species interactions and ecological processes [14].

Many benthic marine organisms spend an early developmental period in the pelagic environment before settling to benthic habitat at the end of this phase [15]. There are a number of sensory cues that are used for navigation and long distance orientation in the marine environment (review by [16]). Auditory cues are valuable as sounds associated with habitat can travel over large spatial scales, and reflect the physical and biological characteristics and quality of the environment [17,18,19,20,21,22,23,24]. There is a growing list of studies that demonstrate that fish, crustacean and other invertebrate larvae orient and settle in response to habitat-related sounds [15,20,24,25,26,27,28,29,30,31,32,33, 34,35,36,37].

Recent studies suggest that ocean acidification might interfere with the ability of fish larvae to detect or respond to ecologically important auditory cues that could be used for habitat selection at settlement. Exposure of fish larvae to high CO_2_ induced changes in the directional response of individuals towards coastal soundscapes in a coral reef species [38], a catadromous species [39] and a temperate species [40]. Despite these similar results, there is also increasing evidence that the effects of elevated CO_2_ on larval behaviour can vary greatly among species [41,42], highlighting the need for further research across a wide range of fish species with contrasting life histories and habitats. Furthermore, these few studies conducted to date [38,39,40] have tested laboratory-reared larvae, but studies on wild larvae are needed since behavioural capabilities may differ between captive reared and wild larvae [43].

In this study we tested the effect of simulated ocean acidification (elevated CO_2_) on the auditory preferences of wild-caught, settlement-stage larvae of a common temperate reef fish species, the painted goby *Pomatochistus pictus*. The painted goby is a small benthic-coastal fish species that inhabits low-turbidity waters in rocky and sandy coastal areas of the Eastern Atlantic Ocean and the Mediterranean Sea [44]. In Portugal the reproductive season ranges from January to May. Males guard eggs in nests through 11–12 days embryonic development [44], after which larvae hatch at approximately 2.8 mm total length, develop in the pelagic environment [45], and then settle into coastal habitat at approximately 17–18 mm [46]. Settlement-stage larvae are usually found schooling close to the substrate (personal observations). Here, wild-caught larvae were exposed for a minimum of 10 days to local ambient conditions (532 μatm ± 58.16, pH 8.06 ± 0.04) and to an elevated *p*CO_2_ condition (1503 μatm ± 71.42, pH 7.66 ± 0.02). The high *p*CO_2_ level was chosen to be close to 1500 μatm, corresponding to a pH decrease of approximately 0.4 units, which is consistent with projections for the end of the century on the current CO_2_ emissions trajectory [47]. Moreover, this species inhabits nearshore regions that already experience *p*CO_2_ levels > 1000 μatm due to upwelling events [48,49], and *p*CO_2_ values up to 1170 μatm have been recorded in the coastal waters where painted goby inhabits [50]. With the amplifying effects of anthropogenic ocean acidification, future *p*CO_2_ could, therefore, easily exceed 1500 μatm. We used an auditory choice chamber to test for responses to night-time recordings of reef sound. If behaviour of settlement-stage larvae was affected by acoustic conditions, we predicted that control (537 μatm) fish would be attracted by these sounds, but that this attraction may be lost in fish reared in high CO_2_ (1503 μatm) conditions.

## Materials and Methods

### Seawater manipulations

Artificial seawater used in the experiments was adjusted to a salinity of 34 psu by blending a commercial salt mixture (Tropic Marin^®^) with filtered freshwater (reverse osmosis system). CO_2_ conditions were maintained by dosing CO_2_ in 200 l sumps to achieve set pH levels. A pH-controller (Tunze Aquarientechnik, Germany) maintained pH at pH_NBS_ 8.0 in the control treatment and pH_NBS_ 7.6 in the high *p*CO_2_ treatment. One sump per *p*CO_2_ treatment was used, each delivering seawater into two replicate 35 l rearing tanks, at ~600 ml min^-1^. Each sump was equipped with biological, mechanical, chemical and ultraviolet filtration. Rearing tanks were sealed with a clear glass lid to limit CO_2_ exchange with the atmosphere. Temperature, salinity and pH in each aquarium were measured twice daily. pH was measured on the National Bureau of Standards (NBS) scale with a portable meter (SevenGo DuoPro, SG23) calibrated weekly with fresh buffers (Mettler Toledo). Oxygen levels were maintained above 90% saturation by the mixing action of the diffusion pumps in the sumps. Samples for determining total alkalinity (TA) were collected from experimental tanks on a weekly basis, placed in air-tight containers without air space, stabilized by mercuric chloride poisoning [51] and kept at +4°C until further analysis. Analyses performed using automated Gran titrations, with certified reference material supplied by A. Dickson (Scripps Institutions of Oceanography, San Diego). *p*CO_2_ was calculated from the *in situ* temperature, TA and pH, using the carbonic acid dissociation constants given by [52] and the CO_2_ solubility coefficient of [53]. Errors associated with *p*CO_2_ calculations were estimated to be ±10 μatm (accumulate errors on TA and pH). Estimated seawater parameters are shown in Table 1.

**Table 1:** Mean (± SD) seawater parameters in the experimental system.

*p*CO_2_ condition	pH_NBS_	T(°C)	S (psu)	TA μmol kg^-1^	*p*CO_2_ μatm
Control	8.06±0.04	16.03±0.28	34.43±0.84	2248.70±11.22	531.97±58.16
High CO_2_	7.66±0.02	16.05±0.23	34.58±0.80	2247.08±4.89	1503.65±71.42

Due to logistical reasons, control water (i.e. not treated with additional CO_2_) was used during all acoustic trials; preliminary tests indicated that there was no difference in behaviour when larvae were tested in control or treatment water. Moreover, a recent study by [54] has shown that predator avoidance behaviour is not altered by experimental test water.

### Larvae

*Pomatochistus pictus* settlement-stage larvae (lacking full squamation) were collected by SCUBA divers at the Arrábida Marine Park (38° 28’ N; 8° 59’ W), Portugal on 14^th^ July and 12^th^ August 2015. Larvae were immediately transported to the laboratory and transferred to four 35 l tanks (~50 fish per tank) with a continuous supply of recirculating seawater, matching field temperature (~ 16°C), and left for one day to recover from transfer and handling. Subsequently, larvae were randomly assigned to two replicate 35 l tanks (~50 fish per tank) per treatment (control or high *p*CO_2_) and reared under these conditions for 10, 15 and 25 days, to test if larvae became acclimated to acidified conditions. The position of the two replicate tanks in control and high CO_2_ treatment was reversed between the capture dates to account for possible confounding effects such as lighting conditions and position in the room. On test days, fish were randomly chosen from each tank/treatment, and used only once. Larvae were reared under locally relevant temperature and salinity conditions, with a summer light cycle of 14h light: 10h dark simulated using fluorescent lights. Larvae were daily fed with *Artemia* nauplii *ad libitum*, with exception of the test day to avoid potential influence of variable recent feeding on performance.

### Fish auditory response

Auditory preferences of larva were tested in acoustic choice chambers [38] using playbacks of recorded reef sound, playbacks of recordings with no biological significance (offshore sound), and control conditions (no playback). Thus, three dual-choice acoustic experiments were performed (fish lengths were measured for inclusion in statistical analyses, see below):

1. reef sound *vs*. no playback (n = 30, standard length 10.36–21.29 mm for control *p*CO_2_; n = 28, standard length 11.13–22.45 mm for high *p*CO_2_);
2. reef sound *vs*. offshore sound (n = 30, standard length 10.45–19.55 mm for control *p*CO_2_; n = 28, standard length 10.16–18.68 mm for high *p*CO_2_);
3. offshore sound *vs*. no playback (n = 29, standard length 10.84–21.58 mm for control *p*CO_2_; n = 27, standard length 10.07–20.93 mm for high *p*CO_2_).

#### Details of acoustic stimuli

Sound recordings were conducted at the Arrábida Marine Park (38° 28’ N; 8° 59’ W), Portugal. Reef sounds were recorded at the same location where *P*. *pictus* larvae were collected. Three reef recordings of 3–4 minutes each were made at dusk (14/07/2015, 20.30 hrs.), in the very nearshore, at a depth of ~10 meters (14/07/2015, 20.30 hrs.); another three offshore recordings of 3–4 minutes each were recorded at 3 miles from the coast, at ~5 meters depth (14/07/2015, 12.00 hrs.). Recordings in both habitats were made under calm conditions and therefore containing few abiotic sounds. Reef recordings were made at dusk, as the biological chorus in most reefs studied so far were found to peak at this time [18,19,55]. The offshore sound was chosen as it is associated with a habitat of low interest for reef-fish larvae, in contrast to reef sounds that represent suitable habitats for settlement and are used as biologically relevant acoustic cues [25,30].

**Fig 1:**
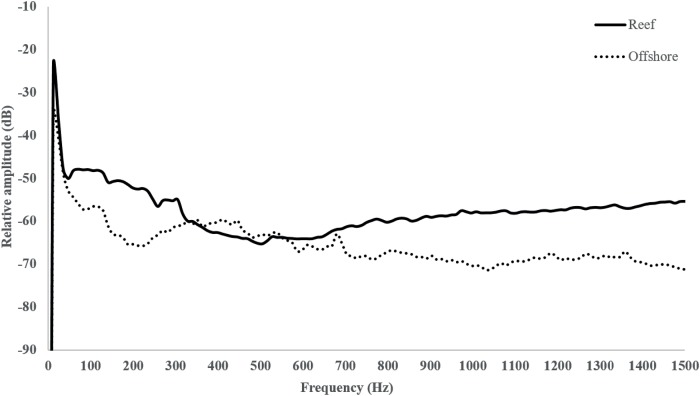
Relative amplitude of reef and offshore sounds (mean of 3 recordings per habitat). Power spectra [fast Fourier transform (FFT), 8192 points, Hamming window].

Sounds were recorded with an omnidirectional hydrophone (HiTech HTI-96-MIN with inbuilt preamplifier, High Tech Inc., Gulfport MS; sensitivity-164.3 dB re 1 V/μPa, frequency range 0.02–30 kHz) connected to a digital Sony PCM-M10 recorder (96 kHz 24-bit Recording, Sony Corporation, Tokyo, Japan). All recordings were made using the same settings so as to allow comparison between their relative amplitudes. Reef sound was on average 12 dB re 1 μPa (RMS) louder than offshore sound (Fig 1).

#### Test protocol

The response of larvae to sounds was tested in an auditory choice chamber (adapted from [38]). The chamber consisted of a transparent acrylic tubular chamber (50 cm long), within which the larva was released, supported inside a glass aquarium (60 x 110 x 25 cm). The tubular chamber had mesh at the two ends, to prevent larvae from escaping. Acoustic stimuli were played back using two underwater speakers (Electrovoice UW-30, Lubell Labs Inc., Columbus, OH, USA), each suspended above the substrate at opposite ends of the aquarium, and kept at a fixed distance of 7 cm from the tubular chamber. To reduce the acoustic resonance and reflections in the auditory choice chamber, aquarium walls were lined on the inside with air-bubble packing film. The aquarium was further insulated from general building noise using layers of ROCKWOOL^™^ and ROOFMATE^™^ placed between the test arena and the research bench. Additionally, each leg of the bench was placed inside a container with sand and ROCKWOOL^™^ to avoid direct contact of the table with the floor.

Sound stimuli consisted of three reef and three offshore recordings, each cut to 3 min of duration. A low-pass filter of 3 kHz was applied to the recordings so that the frequency of sound stimuli was below the resonance frequency of the experimental tank [56] while matching the auditory ability of the species [57].

In each trial the tested sound recording was assigned alternately to the left and right sides of the experimental tank. The sounds files were randomly chosen per *p*CO_2_ condition. Sound stimuli were played back using an audio chain that consisted of two underwater speakers connected to an amplifier (Phoenix Gold QX 4040, Portland, OR, U.S.A.) and fed through D/A device (Edirol UA25, Roland, Osaka, Japan) controlled by Adobe audition 2.0 (Adobe Systems Inc., Mountain View, CA, USA). The average intensity (RMS, full spectra) of sound playback, measured just in front of the speaker, was adjusted to that recorded in the field by regulating the output of the speakers. Electrical noise was reduced by grounding the experimental tank water and all audio equipment.

**Fig 2:**
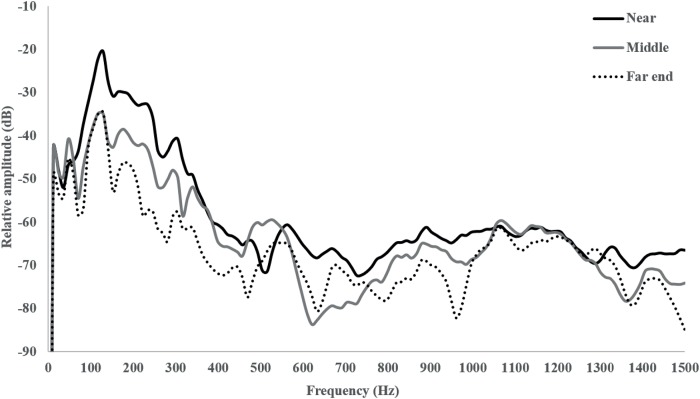
Power spectra of playedback reef sound near the test speaker, in the middle and at the opposite end of the tubular choice chamber. Sound level is higher close to the test speaker, decreasing by 14–15 dB re 1 μPa (RMS) along the chamber.

The average sound playback intensity decreased by approximately 11 dB re 1 μPa (RMS) from the end of the choice chamber near to the speaker to the center of the chamber, and an additional 2–3 dB from the centre to the far end of the tubular chamber (Fig 2). This sound gradient allowed us to test for auditory preferences, as we assumed larvae would spend more time close to the speaker if they experienced a soundscape that they found “attractive”. Ambient conditions in the tank during ‘no playback’ (the silent treatment in some of the experiments) was ~21 dB lower than reef sound playback.

At the beginning of each trial, one larva was placed in a central release chamber in the middle of the auditory choice chamber. We chose to test larvae individually as opposed to groups because behaviour is likely not independent between individuals, and group testing wouldn’t allow us to disentangle the response to a specific sensory cue (sound) from other possible sensorial mechanisms, such as odour or mechanoreception. After 1 min of acclimation, experimental sounds were played for 1 min, after which the larva was released into the tubular chamber, and its position recorded every 5 sec for 2 min. The tubular chamber was divided in half to define the position of the fish at each time interval relative to the playback speaker(s). To avoid changes in water temperatures and olfactory gradients that could influence larval behaviour, the chamber water was renewed between each trial. The sides of the tank with each sound playback was switched regularly to control for unwanted global chamber effects. After each trial, fish were euthanised with an overdose of anaesthetic (MS222 tricaine methane sulphonate; Pharmaq, Norway), fixed in 96% ethanol and measured.

### Statistical analysis

The percentage of time spent by each larvae in the half of the chamber near the test speaker was used as the dependent variable. Percentage data was logit-transformed [log natural(p/[1-p])] for analysis [58]. The ‘test’ speaker was considered to be the one broadcasting reef recordings in reef vs. no playback and reef vs. offshore experiments; and offshore recordings in offshore vs. no playback experiments. Generalized linear mixed models (GLMMs) were used to explore the relationship between the dependent variable and *p*CO_2_ condition, time in treatment and standard length (SL). *p*CO_2_ condition, time and SL were entered as fixed effects, and the tank and capture date entered as random effects to account for multiple fish sampled from the same tank and date. The model that best represented the data set, based on Akaike Information Criterion (AIC), was: Var~ *p*CO_2_ condition + Time + SL + (1|Tank) + (1|Date).

Preference or avoidance for specific auditory cues were tested with one-sample t-tests comparing the percentage of time spent near the test speaker with H_0_ = 0.5, i.e. against a random response of 50%.

All statistical analyzes were done using the R software (version 3.2.2, RFoundation for Statistical Computing, Vienna, Austria) and the lme4 library was used to perform the GLMM analyzes.

### Ethics statement

This study was authorized by the Portuguese National Authority for Animal Health (Direcção-Geral de Alimentação e Veterinária), it was performed in strict accordance with the recommendations of the Animal Care and Use Committee of Ispa-Instituto Universtário, and undertaken under the supervision of an accredited expert in laboratory animal science (following FELASA category C recommendations). Permission for capturing fish at the field site was granted by the National Institute for the Conservation of Nature and Forests (ICNF).

Fish were caught with hand nets, placed into stock tanks, provided with substrate and fresh surface sea water and immediately transported to the laboratory. At the end of the experiment fish were euthanized with an excessive dose of anaesthetics (MS222 tricaine methane sulphonate; Pharmaq, Norway).

## Results

**Fig 3:**
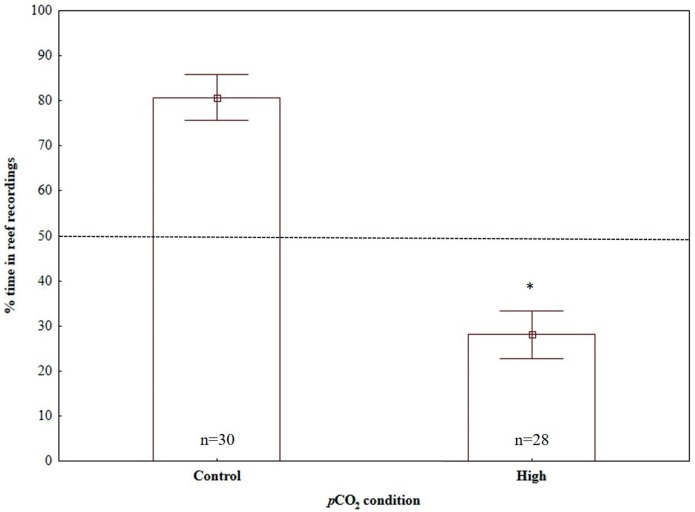
Effects of high CO_2_ conditions on the response of painted goby larvae to acoustic playback of reef sound when tested against offshore sound. Sample sizes are given on bars, which indicate mean ± SE; * indicate significant differences between *p*CO_2_ conditions (p < 0.05).

**Fig 4:**
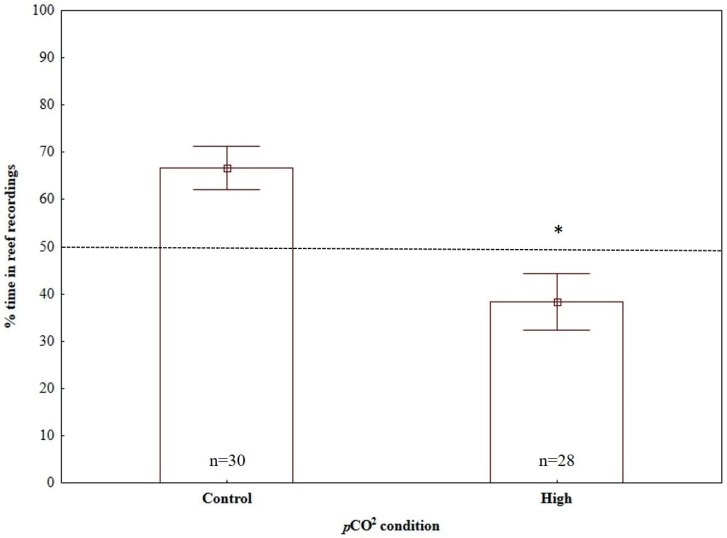
Effects of high CO_2_ conditions on the response of painted goby larvae to acoustic playback of reef sound when tested against no playback. Sample sizes are given on bars, which indicate mean ± SE; * indicate significant differences between *p*CO_2_ conditions (p < 0.05).

**Fig 5:**
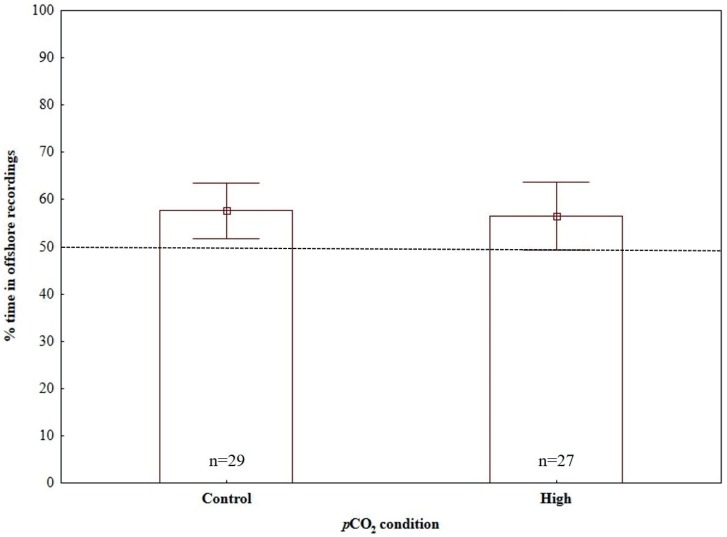
Effects of high CO_2_ conditions on the response of painted goby larvae to acoustic playback of offshore sound when tested against no playback. Sample sizes are given on bars, which indicate mean ± SE.

Time-in-treatment and size did not affect the response of larvae to sound in any of the three dual-choice sound experiments (Table 2). However, a significant effect of *p*CO_2_ condition was detected, with the response of larvae reared in high *p*CO_2_ levels being significantly different to the control reared larvae when experiencing reef recording vs. no playback (Table 2; Fig 3) and reef recording vs. offshore recordings (Table 2; Fig 4); by contrast, no significant *p*CO_2_ condition effect was detected in the experiment of offshore recordings vs. no playback (Table 2; Fig 5).

**Table 2:** Generalized linear mixed-effects (GLMM) model tables for the time spent near the active speaker from larvae maintained at either control or high *p*CO_2_ condition, cross-factored with time in treatment and standard length.

Sound experiment	LME coefficient	SE	df	*t*	*P*
**Reef vs. no playback**					
**Intercept**	1.403	0.992	58	1.415	0.162
***p*CO_2_ condition**	-0.929	0.305	58	-3.041	**0.003**
**Time in treatment**	0.015	0.040	58	0.386	0.701
**Standard length**	-0.017	0.084	58	-0.199	0.843
**Reef vs. offshore**					
**Intercept**	3.522	1.204	57	2.926	**0.005**
***p*CO_2_ condition**	-2.034	0.335	57	-6.077	**< 0.0001**
**Time in treatment**	-0.029	0.041	57	-0.714	0.478
**Standard length**	0.012	0.004	57	0.131	0.896
**Offshore vs. no playback**					
**Intercept**	0.272	1.183	56	0.230	0.819
***p*CO_2_ condition**	0.064	0.359	56	0.178	0.859
**Time in treatment**	-0.023	0.047	56	-0.479	0.634
**Standard length**	0.0172	0.098	56	0.862	0.862

Significance level set at p < 0.05.

Under present-day ambient *p*CO_2_ conditions (control), painted goby larvae showed significant attraction towards reef sound when tested against offshore sound (one-sample t-test, H_0_ = 0.5, t_29_ = 6.08, p < 0.001) and no playback (one-sample t-test, H_0_ = 0.5, t_29_ = 3.65, p = 0.001), spending, on average 80.69% and 66.67% of the time in the half of the chamber close to the test speaker, respectively (Figs 3 and 4). By contrast, no directional auditory response was detected when larvae were presented with offshore recordings vs. no playback (one-sample t-test, H_0_ = 0.5, t_28_ = 1.28, p = 0.21), spending, on average 57.61% of the time close to the half of the chamber broadcasting offshore sounds (Fig 5).

Painted-goby larvae reared in high *p*CO_2_ levels showed an opposite response to control reared larvae, significantly avoiding reef recordings when tested against offshore recordings (one-sample t-test, H_0_ = 0.5, t_27_ = -4.13, p<0.001; Fig 3) and spending less time (although not significant) close to reef recordings when tested against no playback (one-sample t-test, H_0_ = 0.5, t_27_ = -1.93, p = 0.06; Fig 4). However, similar to control reared larvae, no preference or avoidance was detected when larvae were presented with offshore recordings vs. no playback (one-sample t-test, H_0_ = 0.5, t_26_ = 0.91, p = 0.37; Fig 5).

## Discussion

This study demonstrates a strong effect of elevated CO_2_ conditions on the behaviour of wild-caught fish larvae towards suitable settlement habitat cues, by disrupting auditory responses to habitat-specific soundscapes. Underwater soundscapes contain information on habitat features and biological composition that can be used by marine larvae for orientation, habitat selection and settlement [21,22,23,29,30,32,36,59]. Our results show that exposure to high *p*CO_2_ reverses the attraction towards dusk-time reef sounds in wild settlement-stage painted-goby larvae. Larvae exposed to high *p*CO_2_ conditions strongly avoided reef sound, as opposed to larvae in control *p*CO_2_ conditions which were strongly attracted by reef sound. Consistent with our results, settlement-stage larvae of barramundi (*Lates calcarifer*) and mulloway (*Argyrosomus japonicus*) reared in acidified conditions were also repelled from auditory cues from settlement habitat [39,40], and juvenile clownfish (*Amphiprion percula*) showed no preference or actively avoided daytime reef sounds [38]. A note on the experimental set-up is needed here to address the possible issue of pseudoreplication, as the use of a single sump per treatment, feeding replicate tanks, might be considered a pseudoreplicated design (sensu [60]). We argue that pseudoreplication is unlikely to be associated with confounded effects as 1) we have accounted for such issues in our statistical analysis, by using an appropriate multilevel model (GLMM) [61]; 2) we have maintained very high standards of water quality, which made sure there were no other differences in the seawater between treatments, other than carbonate chemistry, that could be responsible for the differences observed; and 3) our results are highly significant, and consistent with other studies [38,39,40], which provides a degree of replication on its own.

In addition to auditory cues, fish use a range of other senses to locate suitable settlement habitat, including olfaction and vision [62]. However, recent studies indicate that these senses are also likely to be impaired by increased CO_2_ [63,64,65,66,67,68]. Moreover, recent evidence suggests that sound production by snapping shrimps, which are among the noisiest invertebrates dominating coastal marine soundscapes, is substantially reduced by exposure to future ocean acidification conditions [69]. This trend towards silence, and the compromised ability to orient towards suitable habitat at settlement may have implications for survival and replenishment of marine populations [70].

Our results also provide evidence that painted goby larvae, from control conditions, are attracted to biologically relevant habitat sounds, but not to sounds deprived of biological significance, such as offshore soundscapes. This agrees with field observations which show that settlement stage larvae of reef fishes orientate away from the reef during the day [71,72,73], but are attracted to nocturnal coastal soundscapes [17,27,29,30,32], presumably due to predation risk being reduced at night [74,75]. Preliminary data on painted goby larvae seem to support this hypothesis, as settlement stage larvae tested in the laboratory avoided day time reef sounds (Faria AM, unpublished results). Reef sounds vary with time of the day and season, and in most studied reefs, biological choruses peak during dusk hours and during the summer time [18,19,55,76,77], coinciding with the arrival of settlement stage larvae in higher densities [78,79]. In general, settlement-stage reef fishes are attracted by the high-frequency sounds of reefs (produced mainly by invertebrates) [80], contrary to juvenile and adult reef fishes that are attracted by low-frequency sounds produced by other fishes [32]. The reef sounds we broadcast in this study contain a mix of frequencies and amplitudes, and we don’t know the components of nocturnal reef sounds to which larvae of our studied species are attracted. Future work could address the sensitivity of this species to different frequencies of sound, using an electrophysiological technique such as the auditory brainstem response (ABR) [38,81], to determine the range of sounds to which this species likely responds. It is also important to note that playback experiments in tanks do not reflect real-world noise sources as the particle motion (the sound component to which larvae are more sensitive) occurs at higher levels in aquaria than in the open ocean [82]. Therefore it would be benifical for future studies to test larval response to natural sounds in their natural habitat. Moreover, the ontogenetic timing of responsiveness towards sound cues should be investigated, as auditory sensitivity and motivation to respond to acoustic cues varies with ontogeneny and among species [39,83]. What our data suggests is that painted goby larvae as small as 10 mm can detect and respond to sound cues, but when, during ontogeny, this capacity develops in unknown; a greater understanding of the species’ hearing abilities will provide valuable information for parameterising larval dispersal models.

The mechanisms responsible for behavioural impairment in fish larvae exposed to high CO_2_ are still uncertain, although it appears to be, at least partially, caused by a disturbance in the GABA-A receptor; the primary inhibitory neurotransmitter receptor in the vertebrate brain [84,85,86]. In the case of auditory sensitivity, changes in otoliths may also account for altered auditory preferences. Otoliths are sensory aragonite structures involved in balance, orientation and sound detection in fishes. However, the few available studies on the effects of acidification on size and shape of otoliths have produced conflicting results—while some species exhibit little effect of near-future CO_2_ levels on otolith development [38,87,88], others show increased otolith growth with increasing CO_2_ [40,89,90], potentially affecting auditory sensitivity [90]. Otoliths of larvae tested in the present study were not analyzed, but previous results of painted goby larvae exposed to CO_2_ levels exceeding the levels used here indicate that neither size nor shape are affected (Faria AM, unpublished results). If otoliths are not affected, the observed altered auditory preference in painted goby under high CO_2_ may be related to altered neurotransmitter function. Future studies should address this hypothesis by treating larvae with an antagonist of GABA-A receptor, such as gabazine [85,86].

The lack of a time-in-treatment effect provides evidence for a lack of acclimation to elevated CO_2_, at least over the time frame of 10–25 days. Consistent with these findings, larvae of a catadromous fish reared from hatching to post-metamorphosis in high CO_2_ conditions did not acclimate to these conditions, despite continuous exposure [40], and juvenile reef fish at natural CO_2_ vents showed similar behavioural disturbances (e.g. bolder behaviour, reversal of olfactory preferences) as those observed in laboratory experiments, indicating that fish did not acclimate despite presumed continuous exposure to elevated CO_2_ since settlement [91]. Futhermore, a review based on short- and long-term experiments and studies at natural CO_2_ vents revealed little evidence of acclimation to acidification for several species [14]. Despite a current lack of evidence for the ability of fish to acclimate to elevated CO_2_ conditions, there remains a need for long-term multigenerational experiments to determine whether species have the capacity to adapt to the predicted ocean acidification over the next century. To date, the few studies on transgenerational acclimation to climate change on fishes yields conflicting results, as some suggest improved growth and survival [92,93], while others suggest that cognitive functions have limited plasticity [94,95]. Determining which traits show transgenerational acclimation, and which mechanisms may be used by species to overcome rapid climate change (including the synergistic effects of ocean acidification and warming), should be a priority for future research.

## Supporting Information

S1 Dataset The file summarizes all the relevant data that have been used in the statistical analyses. (XLSX)
